# Factors Influencing Runner's Choices of Footwear

**DOI:** 10.3389/fspor.2022.829514

**Published:** 2022-03-31

**Authors:** Codi A. Ramsey, Peter Lamb, Daniel Cury Ribeiro

**Affiliations:** ^1^Institute of Sport, Exercise and Health, Otago Polytechnic, Dunedin, New Zealand; ^2^School of Physical Education, Sport and Exercise Sciences, University of Otago, Dunedin, New Zealand; ^3^Centre for Health, Activity and Rehabilitation Research, School of Physiotherapy, University of Otago, Dunedin, New Zealand

**Keywords:** footwear, running, runners, perceptions, running shoe

## Abstract

Until the mid-2000s, qualitative research has been virtually absent from running injury research. A handful of studies have been recently published regarding the attitudes and perceptions of runners and coaches toward injury development. Footwear is frequently perceived as a risk factor for running related injuries, but empeirical evidence fails to support such beliefs. The reasons why runners choose specific footwear warrants formal investigation to further understand the links between footwear and running related injuries. This study aimed to investigate the factors influencing runners choices of footwear. Interviews were conducted with 12 runners. Recordings from the interviews were transcribed verbatim and themes were developed using thematic analysis. Findings revealed 15 unique factors that influence runner's choices of footwear for running. These factors were grouped into three main themes: personal footwear characteristic preferences, other people and economic considerations. Runners largely gather information about their footwear choices from past experiences and people they trust and admire. They also emphasized the complexity of footwear choices due to availability and the constant changes preset within the footwear industry. This research adds to the growing body of knowledge to better understand the wider running injury system. Further studies are needed to establish how runners perceptions of their footwear impact injury rates and to develop effective injury prevention strategies.

## Introduction

There are long-standing debates on how footwear may influence performance, running-related injuries, comfort and participation in running. However, these constructs may be superseded by the runner's behaviors–specifically, their choices of footwear. Recently, it has been suggested that a holistic approach is needed to better understand runner behaviors (Hulme and Finch, 2015). This would allow for more evidence-based, self-management strategies to be developed so that runners—and healthcare professionals and coaches—may cope with individual adaptations to footwear (Malisoux and Theisen, 2020).

In recent years, multiple studies have emerged regarding runners' perceptions of footwear. Data from these studies have been gathered through mostly online questionnaires, which have received thousands of responses from around the world. In general, runners perceive footwear as an important component for comfort (Dinato et al., 2015; Tay et al., 2017; Agresta et al., 2020) and, injury prevention (Saragiotto et al., 2014b; Walton and French, 2016; Fokkema et al., 2017; Dhillon et al., 2020). Additionally, runners have identified that footwear comfort (Dhillon et al., 2020) and matching foot type to shoe type (Saragiotto et al., 2014b) are critical aspects when choosing running footwear. These beliefs may be formed by marketing schemes developed by footwear manufacturers and may not align with scientific evidence. Further analyses have revealed that runners from different countries perceive footwear differently (Kong et al., 2015), perceptions can vary by assessment method (Kong et al., 2015) and performance is not related to runners' perceptions of footwear comfort or prior experience in shoes (Hébert-Losier et al., 2020).

Shoe salespeople's perceptions have been studied in relation to injury prevention. Given the complexities of footwear selection, runners often seek advice regarding which footwear to choose (Walton and French, 2016; Fokkema et al., 2017). Shoe salespeople reportedly feel the work-place training provided by their employers is sufficient to appropriately assess and prescribe running shoes and they believe that running shoes have reduced the incidence of running injuries (Walton and French, 2016). Shoe salespeople's suggestion fit the paradigm that running injury is caused by not matching foot posture to footwear type and believe that expensive shoes are more capable of preventing injury than less expensive shoes (Walton and French, 2016).

Compared to shoe salespeople, physiotherapy students present conservative ratings of their skills and education to prescribe footwear to prevent injury (Walton and French, 2016). Additionally, there is a general lack of consensus whether footwear can prevent injuries (Malisoux and Theisen, 2020) and clinicians lack valid assessment tools to evaluate and prescribe footwear to runners with injuries (Ramsey et al., 2019). Healthcare professionals have found the use of an evidence-based training module helpful for increasing knowledge and perceptions about footwear on injury (Dhillon et al., 2020). However, runners are more influenced by their own internet searches, shoe salespeople and the use of gait in-store assessments, than information from health care professionals (Walton and French, 2016; Dhillon et al., 2020).

Runners may have other preferences and factors that influence their choices of footwear which are not presented in current questionnaire-based research. While previous studies have surveyed large samples of runners to answer questions regarding *what* runners believe, there is a clear gap as to *why* they have these opinions which many surveys do not capture. Additionally, there is a scarcity of qualitative data derived from how runners are impacted by the complexities of footwear selection. This study aims to explore the factors influencing runners' choices of footwear by using a thematic analysis.

## Method

This qualitative study used individual and group interviews to explore the factors influencing runners' choices of footwear. Ethical approval was granted for this study. The 32-item Consolidated criteria for Reporting Qualitative research (COREQ) checklist was used to report this study (Tong et al., 2007). The checklist contains three domains: Domain 1 focuses on research team and reflexivity, Domain 2 focuses on study design, and Domain 3 on analysis and findings which encompass the content and rationale for qualitative research involving interviews or focus group techniques.

### Research Team

All interviews were conducted by the primary researcher, a female PhD candidate, with a background in physical education and a competitive runner. To gain experience with interviewing methods, the primary researcher undertook practice interviews under the guidance of a clinical researcher with qualitative research experience. A second research member, and physiotherapist with extensive qualitative research experience provided quality checking and helped with data coding and analysis. The primary researcher had no prior relationships with the participants included in the study.

### Study Design

#### Theoretical Approach

This qualitative study was guided by a general inductive approach which allows themes to freely emerge from the data (Thomas, 2006). Using thematic analysis allows content-rich data to be interpreted beyond descriptive categories and into themes which can be used to obtain compelling insights into the experiences and issues runners face in real-world settings (Braun and Clarke, 2014). Specifically, a general inductive approach allows factors influencing runners' footwear choices to be explored with flexible guidelines. The social constructivist aspect of this theory permits researchers to interpret theories embedded in the data and include their own beliefs and experiences to bring forward a new understanding of the role of footwear in RRI treatment and prevention. The reasons for conducting the research project were included on the information sheet provided to each participant and the primary researcher's background were stated prior to the commencement of each interview.

#### Participants

Purposive sampling was used to recruit runners from the local community. Runners between the ages of 20 and 55 years old, who, at the time of data collection, were running >30 km per week and were not limited in their training due to injury were recruited for this study. These criteria were chosen to capture healthy recreational runners which are likely to be more experienced with purchasing footwear than novice runners, therefore, providing a richer discussion regarding their footwear choices (Giacomini and Cook, 2000; Booth et al., 2014).

Interested participants contacted the research team via email and were asked to complete a questionnaire that sent electronically via email prior to the interview. The questionnaire included demographic data, training and injury history. Fourteen participants expressed interest in the study and provided informed written consent. No participants dropped out of the study, but interviews with two male runners could not be used due to high levels of background noise. For that reason, those data from those two participants were not included in the analysis of this study. However, it is possible that the content discussed in these interviews may have influenced the researcher's perceptions and subsequently the final data analysis.

Interviews lasted between 20 and 60 min, and no interview was repeated. One interview with a randomly selected participant (Participant H) was conducted with a senior researcher present during the interview. This was done to provide the primary researcher feedback on their interview techniques. Some data from the interview with Participant H was derived from questions from the senior researcher.

### Data Collection

We used a semi-structured, open-ended question interview guide which was adapted during the interview to deepen the data. Prompts were used to collect detailed information about the factors that influence runners' footwear selections. Interview audio was recorded and transcribed verbatim. Field notes were taken during and after each interview and included in the final analysis.

### Data Analysis

The interview transcriptions were read multiple times to familiarize researchers with the content. The primary researcher analyzed all the interviews and arranged data into a coding tree, which was developed in a Microsoft Excel file. Similar text segments from the raw data were grouped together and given a descriptive label (i.e., buy shoes on sale). Labels were then grouped into category descriptions that explain the key characteristics and range of the factors influencing runner's footwear choices (i.e., footwear price). Overarching themes were developed to encompass the impact of the footwear micro-system on runners' choices of footwear. Quotes from the interviews to illustrate the associations to the categories were selected and agreed upon by the research team and are presented in the results. The original texts were cross-referenced to ensure accurate representation within the framework.

Two members of the research team met to discuss emerging themes derived from the data. A second researcher experienced in qualitative methodology, analyzed every second transcript and verified that the labels and categories were appropriate and met the aims of the study. Data saturation was determined when no new labels evolved in two consecutive interviews and was reached by the eighth interview (Guest et al., 2016). Member checks were completed by emailing participants a summary of the categories and interpretation of the interviews. Participants were asked to provide feedback and verify the framework and summary of the results. Any feedback received was incorporated into the final results.

## Results

### Participants

Four female and eight male runners completed interviews for this study. At the time of the interviews, all participants were healthy (not limited in their training by injuries) and running at least 30 km per week (Videbaek et al., 2015). Six runners reported running in more than one brand or style of shoes, depending on the activity or terrain of the training session. All participants' shoes were <1 year old (Table 1).

**Table 1:** Runner characteristics.

**ID**	**Participant type**	**Interview length (min)**	**Sex**	**Age range (years)**	**Experience range (years)**	**Kilometers run (weekly)**	**Footwear**	
							**# in use**	**Age**
A	Recreational runner^*^	27:26	F	46–50	0–5	20–30	1	0–6 months
B	Recreational runner^*^	27:26	F	46–50	10–20	20–30	1	0–6 months
C	Competitive runner	44:27	M	36–40	20+	40+	1+	various
D	Professional runner	44:27	F	31–35	10 −20	40+	1	0–6
E	Competitive runner	16:20	F	36–40	10 −20	30–40	1+	0–6
F	Recreational runner	23:17	M	50+	20+	20–30	1	6–12 months
G	Triathlete	41:14	M	18–25	5–10	40+	1+	various
H	Recreational runner^†^	58:31	M	41–45	0–5	20–30	1+	6–12 months
I	Competitive runner	43:26	M	46–50	20+	40+	1+	various
J	Competitive race walker	37:26	M	50+	10–20	40+	1	0–6 months
K	Competitive runner	35:27	M	41–45	20+	30–40	1	0–6 months
L	Competitive runner	40:36	M	46–50	5 −10	40+	1+	0–6 months

* *group interviews*.

† *senior researcher present*.

### Themes

Three main themes emerged from the interview data indicating that runners' choices in footwear are affected by: economics, other people and personal shoe characteristic preferences. The economic theme emerged as runners expressed concerns and awareness regarding the personal and social costs associated with footwear. Runners explicitly described the other people who they seek information from regarding their footwear choices. Despite the impacts of the first two themes (economics and other people) on runners' footwear choices, it is ultimately their personal preferences that drive the final decision about their shoes. Further analysis of the data allowed the sub-themes to be classified as intrinsic factor vs. extrinsic factors and specified by the main motivations (i.e. injury prevention, performance enhancement or comfort) (Table 2).

**Table 2:** Summary of themes, subthemes and categories.

**Theme**	**Subtheme**	**Category of subtheme**	**Runners supporting sub-theme**
Personal shoe characteristic preference	Brand and model	Extrinsic (Comfort, performance)	B, C, D, E, G, H, I, J, K, L
	Style and specifications	Extrinsic (Performance, injury prevention)	A, C, I, K, L
	Performance	Intrinsic (Performance)	A, B, C, D, E, G, H, I, J, K, L
	Comfort	Intrinsic (Comfort, injury prevention)	A, B, C, D, E, F, H, I, J, K, L
	Modify gait	Intrinsic (Performance Injury prevention)	A, C, E, G, K, L
	Multiple pairs	Extrinsic (Performance, Injury prevention)	C, G, H, J, K, L
Other people	Runners	Extrinsic (Performance, injury prevention)	B, E, F, G, H, I, J, K, L
	Media	Extrinsic (Performance, injury prevention)	B, D, G, H, L
	Salespeople	Extrinsic (Performance, injury prevention)	A, E, C, J, K
	Past experiences	Intrinsic (Comfort, performance, injury prevention)	C, D, E, G, I, J, K, L
	Clinicians	Extrinsic (Injury prevention)	A, B, C, D, E, F, G, H, I, J, K, L
Economics	Cost of shoes	Extrinsic (Performance)	F, H, J
	Availability and selection	Extrinsic (Performance)	C, E, G, I, J, L, K
	Sustainability	Intrinsic (Performance)	E, F, K, L
	Replacement	Intrinsic (Injury prevention)	B, C, D, E, F, H, K, L

#### Theme 1: Personal Preferences

##### Brand and Model

Runners identified aesthetic qualities of footwear that influence their personal preferences. The most discussed extrinsic factor was the brand and model. Runners generally have an affiliation to a specific brand or style of shoe and prefer to use familiar footwear.

“*I've always used [brand - omitted][…], I don't know why, I've never tried any other running shoe”* (Participant F)

Runners expressed being frustrated when models are changed and updated by manufacturers. To avoid this problem, one participant admitted to buying 10 pairs of the same familiar shoe before the manufacturer changed shoe model (Participant I).

##### Style and Specifications

For some runners, their footwear needs to be designed with particular specifications. For example, participants (A, C, I, K, L) discussed choosing shoes based on the amount of heel-toe drop. Other preferred footwear specifications runners often consider when looking for footwear, which include: tread pattern; material of upper, midsole and outer-sole; toe-box width; mass; shoe-lace material; and color. These specifications are preferred for multiple reasons (i.e., personal preference, comfort, performance enhancement, injury reduction/prevention) and is an area where overlapping themes are present. The individual shoe specifications are extrinsic factors by default, but the purposes for them can be translated to the performance and injury prevention sub-categories.

##### Performance

All runners (except Participant F) indicated their level of running performance impacts their footwear choices. Runners who purchase traditional style footwear often reported choosing them for “support”, “shock absorption” (or “cushioning”) during running, while runners who choose minimalist style shoes discussed performance in terms of “power”, “speed”, and “feedback”. There is a common perception that “one day” runners in traditional shoes will be experienced enough to wear minimalist shoes and perform at higher levels. This perception is supported by some experienced runners as Participant L has a sense of freedom and speed in minimalist shoes and Participant I prefers barefoot running to achieve high performance. However, this view is not exclusive to all experienced runners. Participant D feels unnatural in minimalist shoes and has experienced injuries when shoes were too minimal. Furthermore, Participant D feels advantaged by having a custom shoe that is built for performance on the terrain she runs on: “*I can just trust the shoe on everything”* (Participant D)

##### Comfort

Most runners indicated comfort is a primary factor when choosing a new pair of running shoes. Some runners (Participants F, H, J) associated comfort to a specific brand or style of shoe. The parameters defining comfort were different between participants. For example, participants B, C, J, K, and L indicated that comfortable shoes have a wide toe box to accommodate for their foot shape. Four runners (Participants A, B, E, H) assessed comfort by the amount of cushioning in the midsole and believed cushioning to be an important factor in reducing injury risk. Conversely, Participants I, K, and L feel that thick midsoles disconnect them from the ground and make them feel less steady or unable to adjust to uneven terrain. Participant B referred to needing an adjustment period, of sometimes more than a month, before she was completely comfortable in her new shoes, whereas participants C and D could tell immediately whether they would like a shoe. Uncomfortable shoes were considered the cause of injuries for two runners:

“*I could literally walk for a kilometer in a shoe that doesn't suit me, and you know I could be, that tightness could bug me for months”* (Participant C).

“*I just developed calf injury using those [not preferred style of shoe] and I've never felt really supportive as far as running goes”* (Participant K).

##### Modify Gait

Participants A, C, E, G, K, L purchased shoes to promote proper running form, biomechanics or natural gait patterns. Runners reported choosing footwear that is marketed to influence biomechanical components and in-turn, performance. Participant A chose her current shoes because the design is marketed to reduce her foot pronation, which she perceives as negative and indicative of injury. Participants C, I and K purchase shoes with zero millimeters of heel-toe drop to encourage a forefoot strike pattern which they believe provide better biomechanical efficiency during running and prevent injury.

##### Using Multiple Pairs of Shoes

Some runners (Participants C, G, H, J, K, L) rotated through multiple shoes designed for specific activities, events or terrain, (e.g., training shoes vs. racing flats and, trail shoes vs. road shoes). Participant J relies on specialty footwear to achieve the desired performance from using multiple pairs of shoes depending on the task demands. Participants were unable to specifically articulate that injury reduction was the reason they wore multiple shoes but were intuitive toward the underlying principles of variability.

“*I just sense that it's [wearing multiple shoes] not putting my feet through exactly the same kind of function every time. So it's giving them a bit of different movements”* (Participant L)

#### Theme 2: Other People

The term “information gathering” was used by several participants to explain the role other people have on their footwear choices. The personal decision of running footwear is developed through a process of talking to other people and learning from their experiences and opinion, reflecting on past experiences and seeking professional advice.

##### Other Runners

Participants seek advice or information from other runners about shoes regarding the fit, performance, durability, injury protection and costs. Participants (E, G, I, J, K, L) were influenced by other runners to try a new shoe brand or style. One runner (Participant G) has a strong affiliation to a particular brand of shoes because his favorite professional runner wears this brand. Runners view other runners with similar or higher abilities as knowledgeable and trustworthy.

##### Media

Participants were suspicious toward media when shoe reviews were overly optimistic or are released without a long-enough test period. However, trends such as the barefoot/minimalist movement inspired by the book *Born to Run* by Christopher McDougall, and advanced technology “*gimmicks”* are enticing to some runners (Participants B, G, H, L). Participant D indicated that it would be more beneficial to runners if the marketing of footwear was directed toward performance outcomes, rather than designs.

##### Salespeople

Participants (E, C, J) were skeptical of shoe salespeople who do not seem to care about the runner's needs or know about the shoes and are only trying to make a sale. Participant E prefers to buy her shoes online just to avoid being pressured to try on certain shoes by the salespeople. Some runners were also concerned about the use of gait-assessments and other technology used in shoe stores to prescribe a specific type of shoe.

“*I certainly favored the outside of my foot and so they then specified the kind of shoe that would reflect that. I probably found that a little bit too prescribed and formulaic […] so, science in that regard felt more like a gimmick rather than science to me”* (Participant K)

However, Participant A feels the tests and procedures of prescribing shoes has been beneficial and she has been happy with the shoes she has purchased. Runners welcome advice when the salespeople could express their own running experiences and training regimen. Additionally, runners trusted the anecdotes and felt the knowledge of footwear performance and injury prevention qualities was evident when the salesperson could empathize with injury experiences.

##### Past Experiences

Trial and error seemed to be the most reliable source of information gathering among runners (Participants C, D, E, G, I, J, K, L). While trial-and-error is not the influence of another person *per se*, runners reflected upon their past experiences in a way that their footwear choices are influenced by a former version of themselves.

“When I started running a bit more, I went for quite a lot of different shoes, different styles of shoes and these … seem to suit me the best” (Participant G)

##### Clinicians

Some runners rely on footwear advice from people they deem professional, i.e., coaches (Participant C, D, E, J) and physiotherapists (Participants I, K, L). Two participants (K, L) visit a physiotherapist who regularly assesses their footwear for ‘imbalances' and makes necessary adjustments to ensure their shoes are symmetric. These runners are highly influenced by the beliefs of the clinician, perhaps due to the clinicians past experience as a runner, and/or the perceived performance improvements as a result of the clinician's methods.

“*I get my shoes tested quite frequently with a physiotherapist […] he would put it [in-shoe adjustments] in and I could tell quite a big difference […] Without actually being able to feel it or see it but just because of my performance”* (Participant I)

Interestingly, runners who had not experienced an injury severe enough to seek medical attention (Participants A, B, F, G, H) did not indicate that they would speak to a health professional about footwear or injury prevention. Yet, runners who had experienced a past injury and underwent treatment (Participants C, D, E, I, J, K, L) expressed the value of a health professional in their running performance.

#### Theme 3: Economics

##### Cost of Shoes

Runners are mindful of the costs associated with their footwear choices. The price tag can influence whether a runner buys a pair of shoes and is a top concern for (Participants F, H, J). Participants F and H admit they wait for a sale before buying shoes. Some runners indicated that finding shoes online can result in lower costs of footwear, however, they felt guilty for failing to support local businesses. Some runners indicated price is associated to quality and is irrelevant if they believe the shoe limits injury risk. On the other hand, Participant J comments, “*if they were three times the price, I'd still have to buy them, I'm really happy that they're a cheap shoe”*.

##### Availability and Selection

Footwear availability and selection is complicated by the plethora of available models and styles at shoe stores. Runners who are not seeking shoes with specific criteria are overwhelmed (Participants G, I, K, L). Yet, for runners seeking a shoe with certain specifications, there seems to be a shortage in local stores (Participants C, E, J).

##### Sustainability

Some runners' (Participants E, F, K, L) choices of footwear are influenced by an intrinsic concern for the ethical manufacturing and the impact of footwear disposal on the environment. Participants also indicated that they attempt to find ways to recycle footwear through charitable donations of second-hand footwear.

“*I was working in Human Rights and […] One time I actually attended a factory collapse […] and it turned out to be a footwear store and made [running shoes] so it was this massive pile of soles all over the ground outside that had fallen out of this warehouse room and it kinda made me think about what shoes I'm wearing a little bit. How I can avoid that?”* (Participant L)

The awareness of international need for shoes in developing countries impacts when Participant E retires his shoes, as severely worn shoes will be of little use to people in need.

##### Replacement

When considering injury prevention strategies, runners recognized various signals that indicated when to replace their shoes. Some runners (Participants E, F, H) used an intuitive approach and replaced shoes when pain or injury occurred. Participant A admitted to wearing shoes to the point that they were falling apart, just to avoid having to buy another pair of running shoes. She regretted waiting to buy shoes because she was injured for several months and felt if she would have bought shoes sooner, she could have avoided the injury.

Other runners (Participants B, C, D, H) track and monitor the usage and distance of their shoes using running apps on their smart-phones or GPS watches. However, there is some ambiguity about the ideal amount of usage. “*I've got a pair of [shoes] that I think are approaching the use by date because they've met the 500*+ *[kilometer] mark”* (Participant H). Runners also see the end of a shoe's life as an opportunity to try new shoes and perhaps new styles.

## Discussion

The present study explored the factors influencing runners' choices of footwear. Three main themes were revealed in the analysis of the interviews with runners: *(1) Personal footwear characteristic preferences, (2) Other people and, (3) Economics*. The current study has identified several aspects that may be impacting runners' attitudes and behaviors toward footwear. Most notably, runners appear to be highly influenced by the perception that footwear can help them increase performance, alter biomechanics and/or prevent injury. They are persuaded by the anecdotal experiences of other runners and sometimes clinicians or salespeople with similar running goals. This in turn influences the runner to choose shoes that are designed and/or marketed with features or characteristics for specific purposes (i.e., motion control). They also discover the benefits of their preferred shoes through incidental influences such as trial-and-error.

Emerging studies using objective footwear measurements suggest footwear with high flexibility values can alter some biomechanical variables associated with patellofemoral pain (Esculier et al., 2017) but there is no strong evidence that suggests any objective footwear characteristic (heel-toe drop, mass, midsole material, last shape) has protective or causative qualities linked to RRI (Cochrum et al., 2017; Fuller et al., 2017; Hulme et al., 2017a; Zhang and Mcphail, 2017). It is possible that this lack of evidence is because the controlled environment of laboratory-based inquiry requires follow-up of hundreds of participants over several months and few studies have carefully investigated these associations (Malisoux and Theisen, 2020). Additionally, the use of standardized lab footwear—used to reduce bias—may not reflect the runner's real-world choices (Finch, 2006; Hulme et al., 2017b). Even if current research was able to confirm that a specific footwear style or characteristic can prevent (or cause) injury among runners (Malisoux et al., 2016a,b), implementing prevention strategies is problematic due to market competition, health-care developments and, personal preferences and/or experiences.

Comfort is considered a high priority for runners (Willems et al., 2019; Dhillon et al., 2020) and their perception of footwear comfort can impact performance (Luo et al., 2009; Hébert-Losier et al., 2020). Runners may choose footwear consciously based on comfort by comparing multiple pairs against each other (Mündermann et al., 2002) or through a more intuitive approach described as the “comfort filter” (Nigg et al., 2015). Some factors that influence footwear comfort include cushioning, and fit, (Bishop et al., 2020), however these constructs are subjective and runners are not able to reliably assess footwear comfort (Hoerzer et al., 2016). These are also specifications that runners in the current study seek out when choosing their footwear and given that runners are influenced by extrinsic factors (i.e., marketing, healthcare professionals, and other runners), they may choose shoes that are less comfortable because the shoes have the specific features that others prefer or recommend. However, it is unclear whether comfort plays a role in injury prevention and more research in this area is warranted (Malisoux and Theisen, 2020).

Most runners in the present study indicated a level of skepticism toward various information sources. This is contrary to findings from a previous study (Walton and French, 2016). When asked about perceptions toward minimalist and barefoot running, participants in a study by Walton and French (2016) indicated they trust retailers and the internet over medical professionals. Whereas runners in the present study, particularly those using minimalist footwear, were quite vocal about their distrust of marketing and sale tactics such as in-store gait analyses, specifying they lack scientific evidence. This demonstrates that runner's thinking about footwear differ across populations and caution is warranted before generalizing these opinions. As several runners wearing minimalist shoes in the current study depend on the advice and guidance of professionals (i.e., physiotherapists, coaches), it is possible that these runners view physiotherapists and coaches as reliable sources of *current scientific* information. However, it has been shown that physiotherapists lack valid methods of assessing footwear and have beliefs that do not align with current evidence (Richards et al., 2009; Ramsey et al., 2019; Dhillon et al., 2020). The benefits of certain footwear and/or specifications expressed by retailers and shoe review articles may be inflated by profit motives; making navigating the varying opinions and a lack of unbiased evidence problematic for some runners, especially those with fewer years of running experience.

From this study, runners' selection of several pairs of different shoes for specific task-related performances and injury prevention appears to be instinctual and based on common sense. These views are supported by current literature, indicating that parallel use of running shoes is a protective factor from injury among recreational runners (Malisoux et al., 2015). Shoe comfort has been cited as a protective factor against injury (Mundermann et al., 2001), however, running performance is not associated to perceived comfort (Hébert-Losier et al., 2020).

Participants indicated finding the “right” shoe is complicated by the overwhelming selection in stores. This is a paradox supported by literature in consumer behavioral research (Schwartz, 2004; Kinjo and Ebina, 2014), where a purchase can be bypassed due to a high quantity of choices (Kinjo and Ebina, 2014). To evade this paradox, it is suggested that company sale strategies determine an optimal number of products and variety (Iyengar and Lepper, 2000; Kinjo and Ebina, 2014). This involves considering the role of non-purchase behaviors and the consequences associated with the consumers decision-making process (Kinjo and Ebina, 2014). As participants in this study indicated, the constant changes to footwear can result in negative experiences (i.e. costly purchases, reduced performance and/or injury).

As part of a competitive, multi-billion-dollar industry, footwear manufacturers must keep-up with scientific developments and market demand (Barff, 1993; Rixe et al., 2012; Lin et al., 2013). This includes incorporating novel technologies and designs into new shoe models (Barff, 1993). In many countries, footwear import costs are absorbed by the runners who pay higher prices to support local business compared to buying cheaper shoes from overseas or online suppliers (Kelsey, 1999). However, due to the import costs, retailers are careful to only import footwear that will be profitably sold (Kelsey, 1999), limiting the availability of some brands, styles, and sizes–an impact felt by some participants in this study. Retailers should make calculated decisions regarding their stock to find a balance between consumer familiarity and market trends (Kinjo and Ebina, 2014).

### Clinical Implications

Runners in the present study value clinical procedures and advice. The findings of a previous qualitative study, found that runners prefer information from the internet over that of health professionals (Walton and French, 2016). However, runners are likely to only interact with clinicians after experiencing and injury.

Walton and French (2016) suggested health professionals should emphasize their practical knowledge of running and injury prevention to gain credibility among runners (Walton and French, 2016). While credibility is not an issue among the runners in this study, clinicians may contribute to injury prevention by participating in educational forums, completing footwear training modules (Dhillon et al., 2020) and providing advice to novice and non-injured populations. Reiterating the suggestion by d health professionals should emphasize Walton and French (2016), clinicians and footwear specialists should be clear in their instruction and guidance when discussing relative terms such as “comfort”, “support” and “fit” (Walton and French, 2016). This will allow the runner to make informed decisions regarding their footwear and continue to build trust in the clinician.

### Research Implications

It is likely that as novice runners gain experience and become recreational and competitive runners, their knowledge about footwear increases. Through multiple strategies and experiences, runners develop perceptions about how to choose the most appropriate footwear for their needs. Unfortunately, one experience often endured by runners is injury (Van Gent et al., 2007; Buist et al., 2010; Goss and Gross, 2012; Kluitenberg and Van Middelkoop, 2015; Videbaek et al., 2015; Ostermann et al., 2016), therefore increasing their risk of future injury (Saragiotto et al., 2014a; Van Der Worp et al., 2015; Videbaek et al., 2015). Future qualitative inquiry is needed to understand runners' base knowledge of footwear and why they select (or do not select) certain shoes. Through this knowledge, we may be able to establish effective prevention strategies that reduce the risk of *initial* running injury. The link between footwear comfort and performance has been widely researched and has a variety of outcomes (Kong et al., 2015; Agresta et al., 2020; Hébert-Losier et al., 2020; Matthias et al., 2021). Assuming various styles (racing flat vs. traditional/cushioned) would provide different levels of comfort, it could be informative to investigate the relationship of running injuries on the perceived comfort of runners' chosen footwear.

While runners in this study seek advice from physiotherapists, it is perhaps important to underpin the perceptions clinicians have toward footwear in their assessment and treatment of RRI. This would allow a deeper understanding of why some runners rely heavily on clinical advice and footwear monitoring to prevent injury. It may also illustrate alternative methods used in clinical practice that are not yet considered in research objectives.

### Strengths and Limitations

The number of analyzed interviews in this study (n=12) is considered a moderate sample size (Braun and Clarke, 2013) for exploratory qualitative research. More research is needed to explore whether choices of footwear differ by gender and experience levels. We aimed to capture the perspectives of healthy recreational runners; therefore, the perspectives of novice and injured runners are not represented in our analysis.

The data derived from this research is strengthened through dual-researcher analysis. Categories and themes were approved and checked by a second researcher with qualitative research experience. The primary researcher performed a bracketing exercise before commencing interviews. This provides transparency between data derived from participants and the thoughts of the interviewer (Ahern, 1999; Sorsa et al., 2015). Themes, labels and categories were confirmed by a second researcher with varying views to those of the primary researcher, therefore reflecting a balance of opinions. Stake-holder checks were completed by sending all participants a summary of the results to the email they provided on the questionnaire. Participants were encouraged to respond with any comments or suggestions, which were incorporated into the final analysis.

## Conclusion

The present study contributes to an area of footwear research that has previously received little attention. Runners are affected by economic factors and other people when choosing footwear to meet their own needs. Acknowledging the factors influencing runners' choices of footwear indicates that runners may engage in behaviors that contribute toward injury prevention and performance enhancement. However, it remains unclear whether runners' choices of footwear are actually linked to injury or performance. Further research is needed to gain a deeper understanding of the complex running-injury system (Hulme et al., 2017b) and to developing appropriate injury prevention strategies.

## Data Availability Statement

The raw data supporting the conclusions of this article will be made available by the authors, without undue reservation.

## Ethics Statement

The studies involving human participants were reviewed and approved by University of Otago Human Ethics Committee. The patients/participants provided their written informed consent to participate in this study.

## Author Contributions

CR collected and analyzed the date and wrote the manuscript draft. All authors contributed to the design of the study, provided feedback, and approved the final version.

## Funding

CR was supported with a doctoral scholarship during the data collection of this study. This research was conducted during tenure of The Sir Charles Hercus Health Research Fellowship of the Health Research Council of New Zealand (Grant number: 18/111) awarded to DCR. The Health Research Council - New Zealand had no role in the design of the study, its execution, data analysis, and interpretation or on the submission of the manuscript for publication.

## Conflict of Interest

The authors declare that the research was conducted in the absence of any commercial or financial relationships that could be construed as a potential conflict of interest.

## Publisher's Note

All claims expressed in this article are solely those of the authors and do not necessarily represent those of their affiliated organizations, or those of the publisher, the editors and the reviewers. Any product that may be evaluated in this article, or claim that may be made by its manufacturer, is not guaranteed or endorsed by the publisher.
